# Supplementary material for: Genetic and Molecular Evidence of a Tetrapolar Mating System in the Edible Mushroom Grifola frondosa
Source: J Fungi (Basel). 2023 Sep 23;9(10):959. doi: 10.3390/jof9100959 (PMC10607315; doi:10.3390/jof9100959)
Supplement: Supplementary file 1 [file jof-09-00959-s001.zip › jof-2552570-supplementary 1.pdf]

Genetic and Molecular Evidence for a Tetrapolar Mating System in the Edible Mushroom, *Grifola frondosa*

Shuang-Shuang Zhang [1,†], Xiao Li [1,2,3,†], Guo-Jie Li [1,2,3], Qi Huang [1], Jing-Hua Tian [1,2,3], Jun-Ling Wang [4], Ming Li [1,2,3] and Shou-Mian Li [1,2,3,*]

[1] College of Horticulture, Hebei Agricultural University, Baoding 071001, China; 13722717690@163.com (S.-S.Z.); lixiao@hebau.edu.cn (X.L.); liguojie.imcas@foxmail.com (G.-J.L.); huang75661@163.com (Q.H.); yytjh@hebau.edu.cn (J.-H.T.); yyliming@hebau.edu.cn (M.L.); yylsm@hebau.edu.cn (S.-M.L.)

[2] Hebei Key Laboratory of Vegetable Germplasm Innovation and Utilization, Baoding 071001, China; lixiao@hebau.edu.cn (X.L.); yyliming@hebau.edu.cn (M.L.); liguojie.imcas@foxmail.com (G.-J.L.); yytjh@hebau.edu.cn (J.-H.T.); yylsm@hebau.edu.cn (S.-M.L.)

[3] Collaborative Innovation Center of Vegetable Industry of Hebei Province, Baoding 071001, China; lixiao@hebau.edu.cn (X.L.); yyliming@hebau.edu.cn (M.L.); liguojie.imcas@foxmail.com (G.-J.L.); yytjh@hebau.edu.cn (J.-H.T.); yylsm@hebau.edu.cn (S.-M.L.)

[4] College of Life Science, Hebei Agricultural University, Baoding 071001, China; wangjunling2001@163.com (J.-L.W.)

* Correspondence: yylsm@hebau.edu.cn (S.-M.L.)

† These authors contributed equally to this work.

Supplementary Material

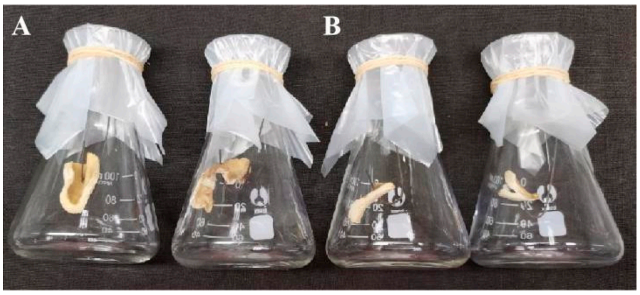

Supplementary Figure S1. Collecting basidiospores of *Grifola frondosa* dikaryotic strains LMXY (**A**) and LMCZ (**B**).

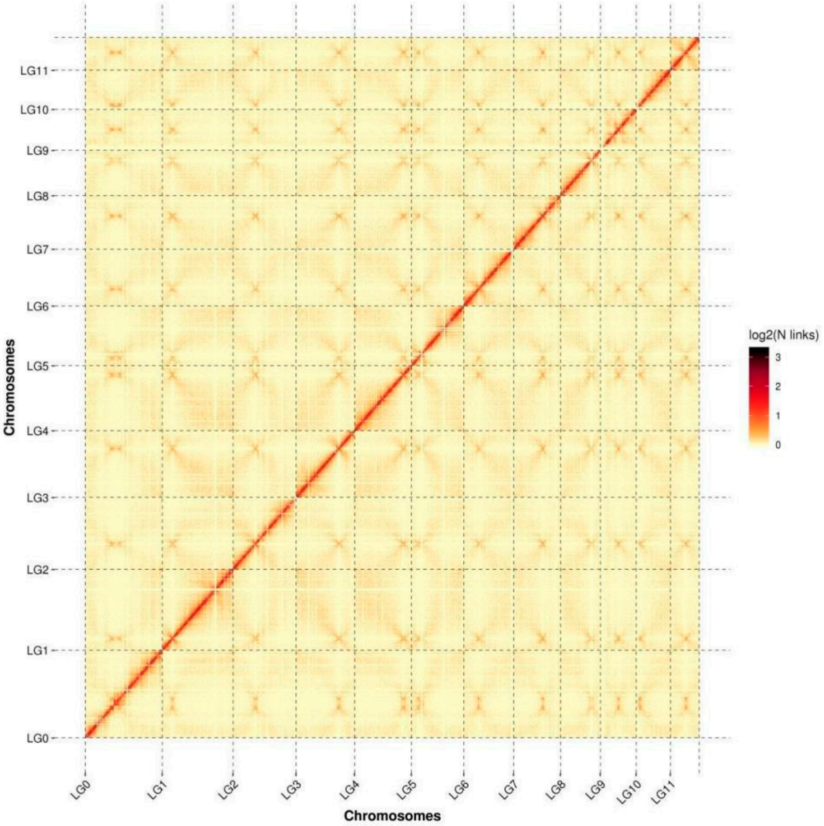

Supplementary Figure S2. Intensity signal heat map of the Hi-C chromosome.

Note: 'Lachesis Group' (LG) stands for the chromosome.

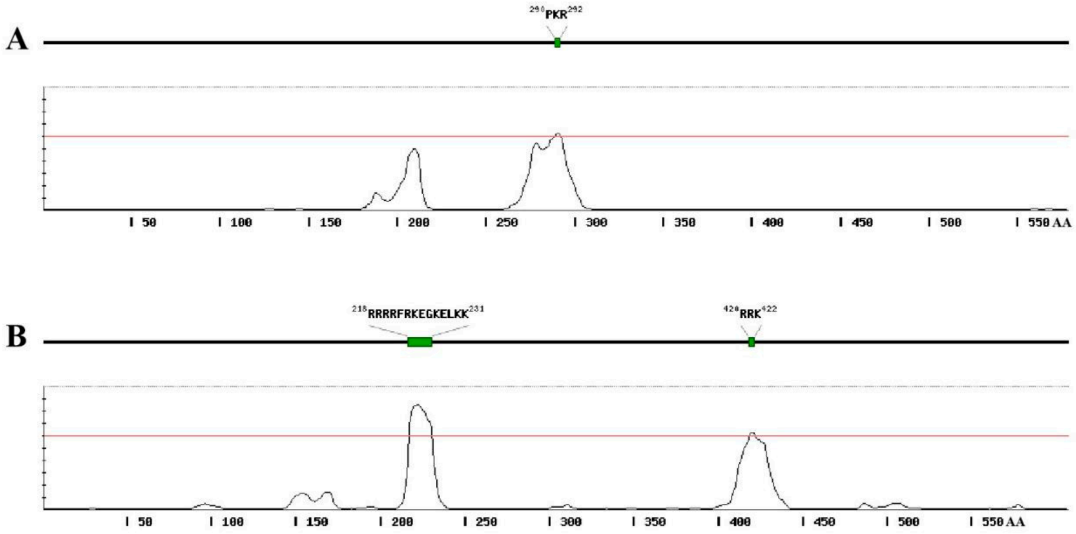

Supplementary Figure S3. Nuclear localization signal analysis of HD1 (**A**) and HD2 (**B**).

Note: Horizontal red line depicts the chosen posterior threshold of 0.6.

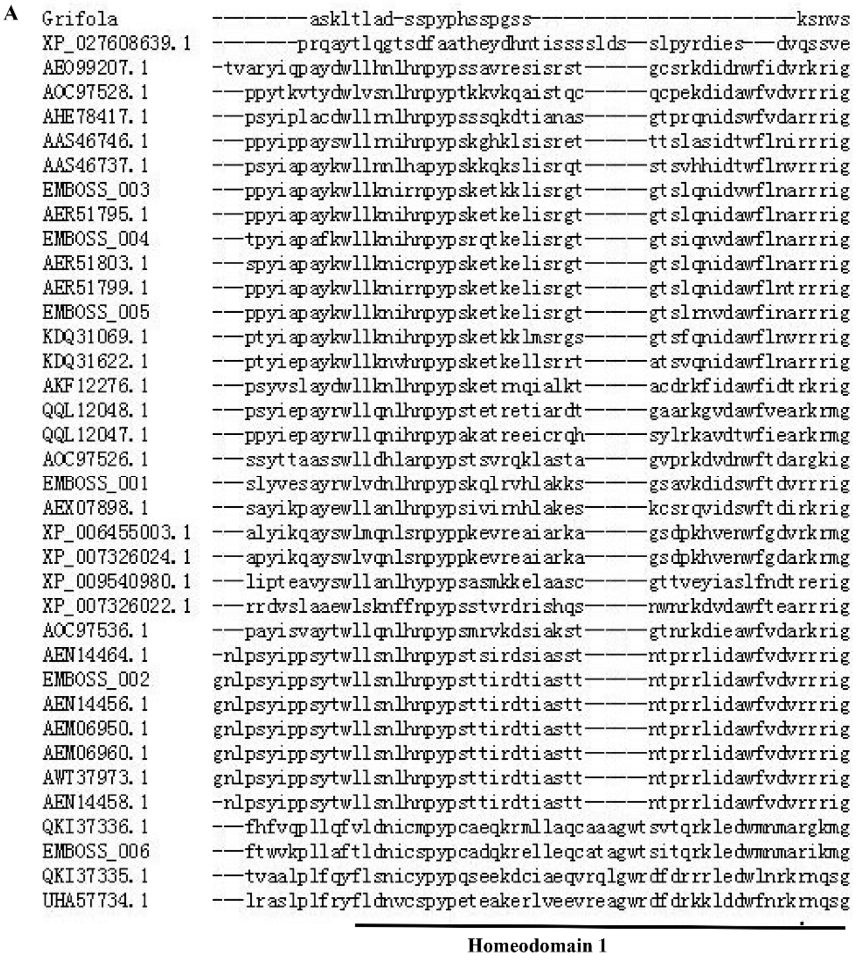

B

```
Grifola            --------EIKVRALRAKQRQIEVLNA--APKHPRRTFSQ-----------HVISILEK-
XP_027608640.1     --------esrldelrsrnfnet------srkntrlpfnq-----------gaipilek-
AOC97529.1         ---------lrlasqytqqld--------ssptsrpsfkt-----------eftpller-
AOC97527.1         --------efmlshpsgkkas----------senrsqfnq-----------nsvpmlek-
AAS46736.1         --------ALHLDKLQADQVK----------PAAKTSFNA-----------DFVPFLEK-
AAS46747.1         --------VLHQQKVKGDKLK----------PATKIVFNA-----------TFVPFLEK-
UWI70524.1         --------rahceklrssvqs----------rpsknafnh-----------dyvsvlek-
UWI70522.1         --------sshceklrssrqssak-----gnrsvktvfnh-----------dyvpvlek-
UWI70526.1         --------rqqcdklrsn-ptpvq-----tktstkasfny-----------dyapilek-
AER51796.1         --------qeqceklrneavkltk-----a-kqakpgfnh-----------dyvpvlek-
AER51800.1         --------kiqcdqwrtavttha--------kpakaafnh-----------dfvpvlek-
KDQ31068.1         --------kvrcdrlreqa------------kpkkpsfnh-----------dyvpvlek-
AER51804.1         --------qlrcdvdttktakass-----s-rptktvfnytlaayerllcqdyvpvlek-
AKF12275.1         --------rvavdqakklqqmqs--------tkpkpifnq-----------efvpllet-
QQL12046.1         --------raflatvvpevdasn------itkekkpvfnh-----------eytpllek-
QQL12049.1         --------elaidrvrgaqgt---hdadq--pakrqtfnq-----------efvpllek-
EKM84450.1         --------arrlrknrgiep-----------ttckpsfky-----------eyvpllns-
AEX07899.1         --------LSGIDT-----------------RSKNPPFNH-----------AYTPILEK-
AOC97531.1         ---------tklsk---------------qyvqdrksfnq-----------eyipylmk-
AEX07900.1         --------ESYVATTTPASISSEK-----QERKERLPFKA-----------EFTPLLEN-
AEO99208.1         --------RKRVDYILTSIISSH--------DRKRKAFNH-----------HYSHLLER-
AOC97535.1         --------rkrvdyiltsiissh--------drkrkafnh-----------hyshller-
AHE78418.1         --------EAWIESARAKRRVSK-----DENSTQRPRFKH-----------EYSQLLEN-
AOC97533.1         --------eawiesarakrrvsk-----denstqrprfkh-----------eysqllen-
AEN14465.1         --------LNGLETYAKEHPPHP--------STPPPAPRQ-----------TSIPAYEAP
AEN14463.1         --------mtevetfakeh-prp--------stpppaprq-----------ssipayeap
AEM06955.1         --------mtevetfakeh-prp--------stpppaprq-----------ssipayeap
AEM06957.1         --------mtevetfakeh-prp--------stpppaprq-----------ssipayeap
AEM06959.1         --------mtevetfakeh-prp--------stpppaprq-----------ssipayeap
AEN14461.1         --------vaqvevfak--------------stpppptrq-----------ssipayeap
AEM06951.1         --------vaqvevfak--------------stpppptrq-----------ssipayeap
AEM06953.1         --------vaqvevfak--------------stpppptrq-----------ssipayeap
AEN14459.1         --------leevkayaenh-prp--------stpppptrq-----------tsipayeap
AEM06961.1         --------meavkehaekh-prp--------stpppptrq-----------ssipayeap
AEM06963.1         --------meavkehaekh-prp--------stpppptrq-----------ssipayeap
AEM06964.1         --------meavkehaekh-prp--------stpppptrq-----------ssipayeap
AEN14457.1         --------ieavkehaekh-prp--------stpppptrq-----------ssipayeap
AWT37972.1         --------meavkqyaeehpprp--------stpppptrq-----------ssipayeap
```

Homeodomain 2

```
Grifola            -------------FFERNAFPSRSEKQGLAAET---NMTYTQVHVWFQNRRRRFR-KEGK
XP_027608640.1     -------------ffernafpsraekeqlasia---dmdykqiniwfqnrrsrsk-kqgk
AOC97529.1         -------------yfaqnaypsapdrttlarkt---gmtirqievwfqnhrnrak-rngk
AOC97527.1         -------------yfqqnaypsaadrvalakrs---smtprqievwfqnhrnrar-kegk
AAS46736.1         -------------YFEFNAYPSAADRSLMARKS---MMTPRQIEVWFQNHRNRAR-KEGK
AAS46747.1         -------------YFEYNAYPSAADRALMARNS---MMTSRQIEVWFQNHRNRAR-KEGK
UWI70524.1         -------------yfeynaypsaadralmarks---mmterqievwfqnhrnrar-kdgk
UWI70522.1         -------------yfeynayptaadralmarks---lmterqievwfqnhrnrar-kegk
UWI70526.1         -------------yfeynaypsvadralmarks---mmterqievwfqnhrnrar-kdgk
AER51796.1         -------------yfecnaypstadralmarks---mmterqievwfqnhrnrar-kegk
AER51800.1         -------------yfeynaypsaadraliarks---mmterqievwfqnhrnrar-kdgk
KDQ31068.1         -------------yfeynaypsaadrtlmarks---mmtdrqievwfqnhrnrsr-kdgk
AER51804.1         -------------yfeynaypsaadralmarks---mmterqievwfqnhrnrar-kdgk
AKF12275.1         -------------yfeynaypsapdraslakks---mmttrqievwfqnhrnrak-kegr
QQL12046.1         -------------yfeynaypsardravlarks---mmtqrqievwfqnhrnrar-kdgk
QQL12049.1         -------------yfeynaypsapdrtalarks---mmtprqievwfqnhrnrak-kegk
EKM84450.1         -------------yfkrnaypstsdraslakkt---nmssrqievwfqnhrnrah-kngq
AEX07899.1         -------------YFESNAYPSAADRAVLARKS---DMTPRQIEVWFQNHRTRAK-KEGR
AOC97531.1         -------------yfeqnpypstadrallarks---gmsprqievwfqnrrnrak-rdgr
AEX07900.1         -------------YFAKNAYPSSADRTVLAKKS---GMTQRQIEVWFQNHRNRAK-KEGR
AEO99208.1         -------------YFEQNAYPSAADRRHLAQKT---VMSPRQIEVWFQNHRSRAR-KEGK
AOC97535.1         -------------yfeqnaypsaadrrhlaqkt---vmsprqievwfqnhrsrar-kegk
AHE78418.1         -------------YFSKNAYPSRPDRAVLARKS---GMTLKQIEVWFQNHRSRAK-KEGR
AOC97533.1         -------------yfsknaypsrpdravlarks---gmtlkqievwfqnhrsrak-kegr
AEN14465.1         VPFNNEYTPILETYFQYDPYPTSRDRQIIAERS---GMTRRQIEVWFQNHRRTAR-QSGI
AEN14463.1         vpfnneytpiletyfqydpyptsrdrqiiaers---gmtrrqievwfqnhrrtar-qsgi
AEM06955.1         vpfnneytpiletyfqydpyptsrdrqiiaers---gmtrrqievwfqnhrrtar-qsgi
AEM06957.1         vpfnneytpiletyfqydpyptsrdrqiiaers---gmtrrqievwfqnhrrtar-qsgi
AEM06959.1         vpfnneytpiletyfqydpyptsrdrqiiaers---gmtrrqievwfqnhrrtar-qsgi
AEN14461.1         vpfnneytpiletyfqydpyptsrdrqiiaers---gmtrrqievwfqnhrrtar-qsgi
AEM06951.1         vpfnneytpiletyfqydpyptsrdrqiiaers---gmtrrqievwfqnhrrtar-qsgi
AEM06953.1         vpfnneytpiletyfqydpyptsrdrqiiaers---gmtrrqievwfqnhrrtar-qsgi
AEN14459.1         vpfnneytpiletyfqydpyptsrdrqiiaers---gmtrrqievwfqnhrrtar-qsgi
AEM06961.1         vpfnneytpiletyfqydpyptsrdrqiiaers---gmtrrqievwfqnhrrtar-qsgi
AEM06963.1         vpfnneytpiletyfqydpyptsrdrqiiaers---gmtrrqievwfqnhrrtar-qsgi
AEM06964.1         vpfnneytpiletyfqydpyptsrdrqiiaers---gmtrrqievwfqnhrrtar-qsgi
AEN14457.1         vpfnneytpiletyfqydpyptsrdrqiiaers---gmtrrqievwfqnhrrtar-qsgi
AWT37972.1         vpfnneytpiletyfqydpyptsrdrqiiaers---gmtrrqievwfqnhrrtar-qsgi
```

Homeodomain 2

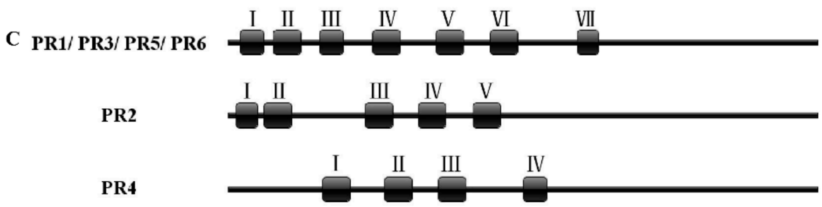


```
PR4  -----------------------------------------MLWTSIACLNQFINSIVWA
PR5  ---MADPTYPLFPVFAFLAFILTLIPLPWHLQAWNSATCFYMFWASIASLNEFVNSVVWA
PR1  MAIPWEVQGEIYAAFALLGFILVCIPLYWHMEAWNVGCVLYIFFVGTECLIKFINTIVWR 60
PR3  -------MHPELPYIAFLAAFLVLIPLPWHWRAGNFATVSIIAWLFVVNVIYGVDAIIWH
PR6  ------MYYPALPAGAFIAAVFVLIPIPAHWRARNVATLSIIVWLFVMDIIYGVNAIVWD
PR2  -------MRAELPVISFLCVVLLITLAPLHWHSRNVAVLSIIIWLLICNIIQGINTIVWA

PR4  DNAINFAPVWCDISTRIIIGASVGIPASSLCITRRLYKISRLQAVVVTRAEKGRMVLIDS
PR5  SNALNPAPVWCDISTRIMIGASVGIPAASLCINRRLYIIACVQTATITKPEKLRAVLIDS
PR1  NNAINWAPVYCDITTRFRLAVSIGVCTASLIINRRLYKIATVSSVSISRSDKRRAVLVDC 120
PR3  GNVAIVVPVWCDITTKIMIGANIALPAACMCVCIHLEQVASVRRVRTMLSDKRRRQIFEA
PR6  DSVEIHMLVWCDITTKIAIGASTALPAAVMCVCKHLELVASGRVVRLTSDDKRRRMIFEL
PR2  ENAVVRIPVWCDIVTKLLLGARIALSAASLGLCHNLRNSISVTETKG----RRHAFTLDL

PR4  LLCVLLPIIYIALEYIVQGHRFNIFENLGCYPAIYNTLPAYFLVYMWPVAIGLVSSIYCG
PR5  LVCLLFPILVIALSYIVQGHRFNIFEDIGCFPAIYNTLLAYFLVYWWPLVLGLISAVYCI
PR1  AIGLGIPVLAVILYWFYQGHRFDILEGIGCIPAIPNTILSYFLYEAWPIPIGLVSAFYCI 180
PR3  VICFLVPMLWMGLHYTVQGHRFDIIEDYGCRPNTYVSIPSIFLIWVPPLIFSAVTFVYAA
PR6  IMCYVIPAVFMALHYVVQGHRFDIIQFFGCQPATYYSIPAVFIVWFPPLLFSVIASFYAA
PR2  VINVTLPFIYMALHAIVQARRFDLAENLGCDASTYSSVPAIILVWLPPLIFCIATLIYAC

PR4  LALRALLQRQAQFREFLSHG-SSLTVSRYLRLMALAMTEILFTIPLGVYELYSNAIGGAL
PR5  LSLRSFLKRRVQFNQLLASKQSSLTMGRYFRLMALATTELLLTVPISTYAIYLNATAQPV
PR1  STIRAFLKRRKQFNELMASN-SNLTFNRYLRLMALASADMLFTVPLGIYSIVSNLKANPI 240
PR3  VAFMHFFRRRITFAKHLENSRAGLTTSRYLRLMAMSIVEMVISAAATSTTLWFTTLT--L
PR6  FALYHFFRQRFIFSMRLQSSNSALSTSRYLRLIAMSVTEIIWQTALTALVMYDNISPG-L
PR2  FILFTWFKRHSLFNNHAIDTSRLSVFTILRPLLTASLIASIIIASTAFSMYARIATSGGL

PR4  QPWKGWDDTHFNFSHVGQYIAAVWRADPTLRIPLELSRWIVVVCAFVFFGFFGLAKDARR
PR5  GPWRSWADTHFAFARVDQIPDLIWRMDHDIVVAQELGRWLSPVCGLIFFAYFGFASEARR
PR1  YVWRGLADIHFGFSRVEQMPSFVWRQSPAMVQAMNFDNWMIVACAFLFFALFGLAEEART 300
PR3  RPWTNWADVHWNFSRVDVYFTAFTPR--FVLVYYYIAWLILPISSLIFFLFFAFGQDALQ
PR6  RPWTNWADVHSNFSRVGQFFILEFMP--SYQRQFFLFFWVMPISSFVFFIFFGFGEETMK
PR2  LSWTSLYQVHAQFSQVNIIP----QSSRLYLAQLEFTWWTIPACSLVLIAMSLPGLASRE
```

Supplementary Figure S4. Amino acid sequence alignments of HD1 (**A**), HD2 (**B**) and PR (**C**).

Note: A and B show parts of the alignment of HD1 and HD2, respectively; black boxes and red high-lightings in C indicate approximate organization of transmembrane helix structures.

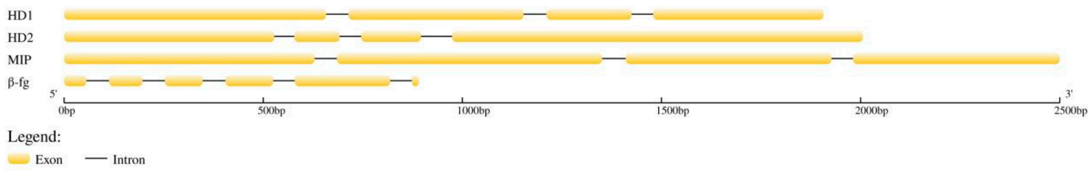

Supplementary Figure S5. Structures of genes at Mate A loci.

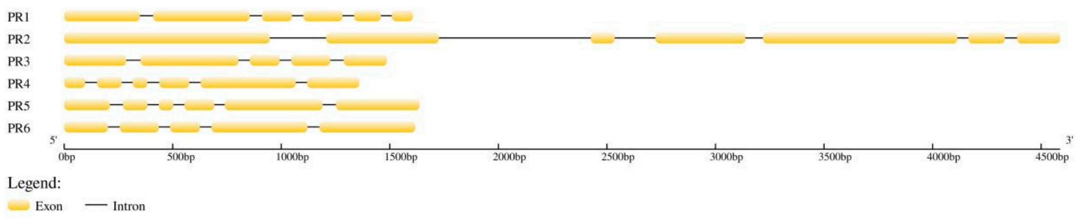

Supplementary Figure S6. Structures of the six pheromone receptor genes.

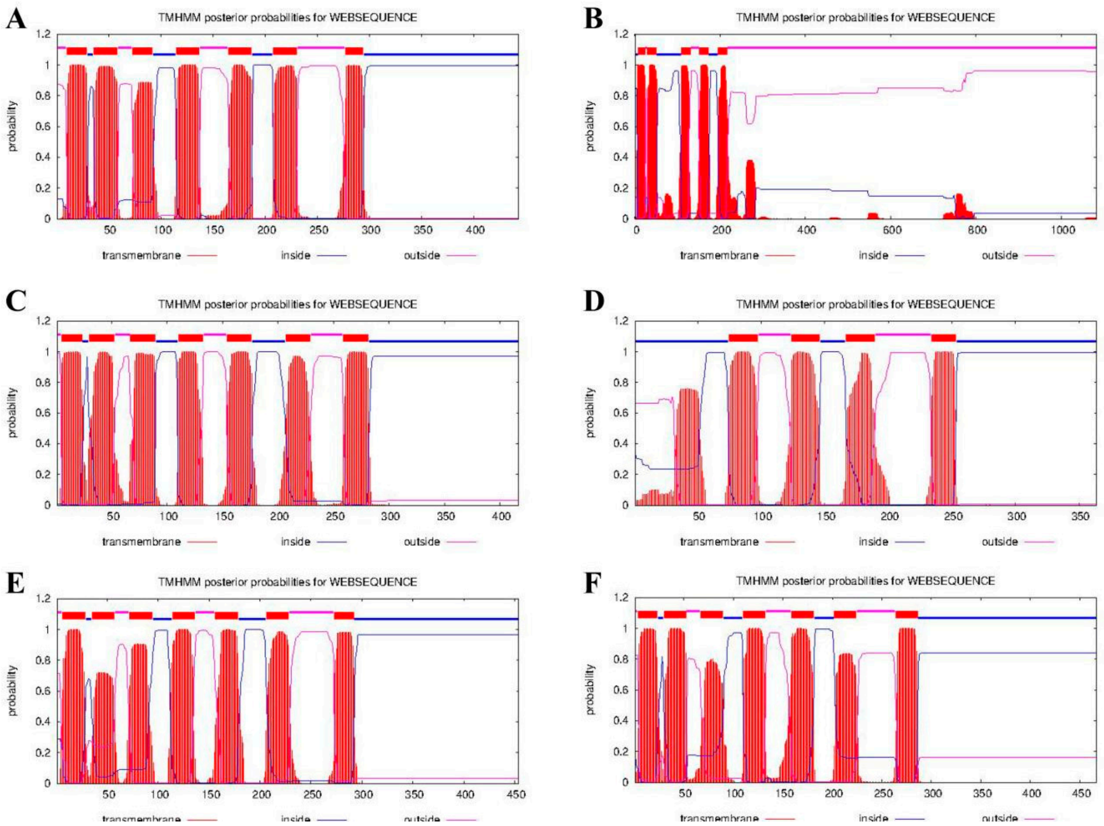

Supplementary Figure S7. Transmembrane structure of the six pheromone receptors.A: PR1; B: PR2; C: PR3; D: PR4; E: PR5; F: PR6.

>PP1

MSDSTSAISTNSTKCSNTYVHCLLATVLCRSPAQMNASFAPVLQLITEPATYSSMSIKIR

>PP2

MIPRIFILCVLFSSTARFVKCGTVVDGMVELMTCDSPSEPLAEQCVSACQPVRDSQVCGQKAACTCAAAP
PAAVWVCMQCQFHTAQDAREPGSAFIVTLRMNAYSRLCGEPLVDETITSALGVSASQERNTTVKMRELP
GICFYGLPEVTIVDRPAASSGRFGLATYGLFFSVFVMAVVMYEL

>PP3

MDDIFTPITIIEEEDHEGPSIPVDEDHPFNFPAYCVIAACSSDWRVPDTRTFGWYDGFLILERDSDWSSWP

>PP4

MDEFSQLINEFSELVDGVSQLVALPRVVTRIGGDDGVPVNEDSISRAGIYCIIGVRSNLPFPSLSNLSLI

MQQFVCGISYRVLLYLPYH

>PP5

MVGTPYWMAPEVVKQKEYGAKALYLIATNGTPTLKKPEALSRELKGFLSVCLCVDVKSRATADELLEHEFLKKACALSGLAPLLRFKNKQAS

Supplementary Figure S8. The amino acid sequences of pheromone precursor proteins.

Note: The conserved sequences are highlighted in yellow.

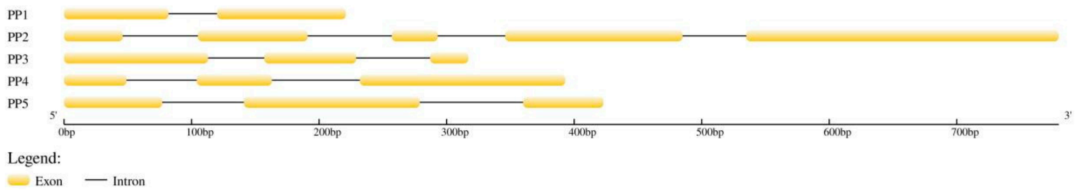

Supplementary Figure S9. Gene structures of five pheromone precursor genes.

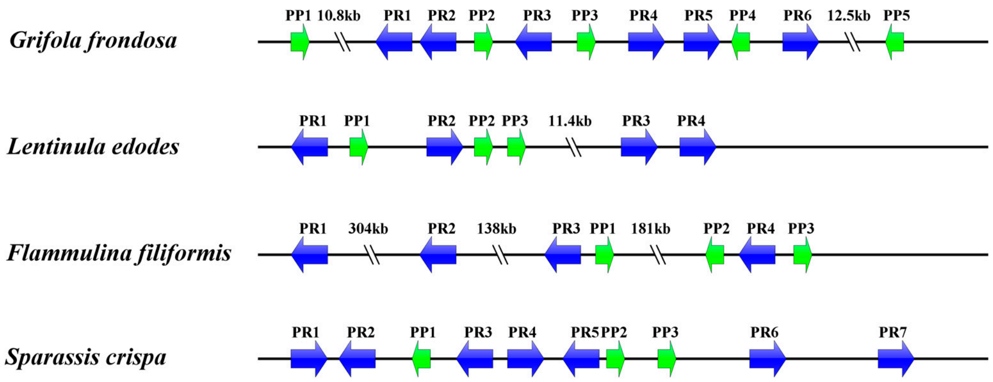

Supplementary Figure S10. Synteny around the pheromone receptors and pheromone precursors of some edible fungi.

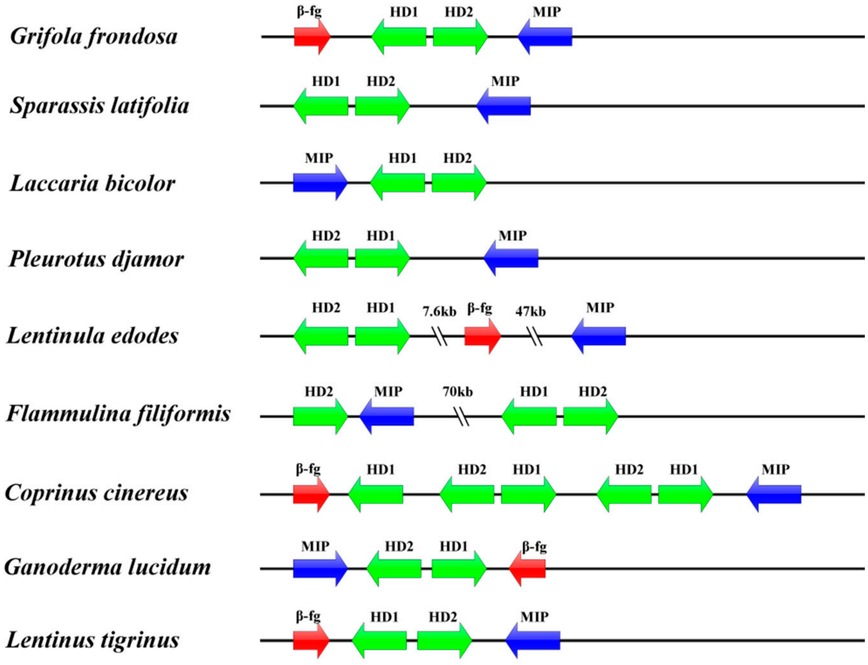

Supplementary Figure S11. Synteny around the HD locus of some edible fungi.
